# Comprehensive Analysis of DNA Methylation and Prediction of Response to NeoadjuvantTherapy in Locally Advanced Rectal Cancer

**DOI:** 10.3390/cancers12113079

**Published:** 2020-10-22

**Authors:** Luisa Matos do Canto, Mateus Camargo Barros-Filho, Cláudia Aparecida Rainho, Diogo Marinho, Bruna Elisa Catin Kupper, Maria Dirlei Ferreira de Souza Begnami, Cristovam Scapulatempo-Neto, Birgitte Mayland Havelund, Jan Lindebjerg, Fabio Albuquerque Marchi, Jan Baumbach, Samuel Aguiar, Silvia Regina Rogatto

**Affiliations:** 1 Department of Clinical Genetics, University Hospital of Southern Denmark, 7100 Vejle, Denmark; luisa.matos.do.canto.alvim@rsyd.dk; 2 International Research Center–CIPE, A.C. Camargo Cancer Center, Sao Paulo 04002-010, Brazil; mfilho@accamargo.org.br (M.C.B.-F.); fabio.marchi@accamargo.org.br (F.A.M.); 3 Department of Head and Neck Surgery, Hospital das Clinicas HCFMUSP, Sao Paulo 01246-903, Brazil; 4 Department of Chemical and Biological Sciences, Institute of Biosciences, Sao Paulo State University (Unesp), Botucatu 18618-689, Brazil; claudia.rainho@unesp.br; 5 Institute of Biological Psychiatry, Psykiatrisk Center Sct. Hans, 4000 Roskilde, Denmark; diogo.marinho@gmail.com; 6 Colorectal Cancer Service, A.C. Camargo Cancer Center, Sao Paulo 04002-010, Brazil; bruna.catin@accamargo.org.br (B.E.C.K.); samuel.aguiar@accamargo.org.br (S.A.J.); 7 Department of Pathology, Sírio-Libanês Hospital, Sao Paulo 01308-050, Brazil; maria.dfsbegnami@hsl.org.br; 8 Molecular Oncology Research Center, Barretos – 14784-400, and Diagnósticos da América (DASA), Barueri 06455010, Brazil; cristovam.neto.ext@dasa.com.br; 9 Department of Oncology, University Hospital of Southern Denmark, 7100 Vejle, Denmark; Birgitte.Mayland.Havelund@rsyd.dk; 10 Danish Colorectal Cancer Center South, 7100 Vejle, Denmark; jan.lindebjerg@rsyd.dk; 11 Department of Pathology, University Hospital of Southern Denmark, 7100 Vejle, Denmark; 12 TUM School of Life Sciences Weihenstephan, Technical University of Munich, 85354 Freising, Germany; jan.baumbach@wzw.tum.de; 13 Institute of Regional Health Research, Faculty of Health Sciences, University of Southern Denmark, 5000 Odense, Denmark

**Keywords:** 5-fluorouracil, predictive biomarker, high-throughput DNA methylation analysis, translational research

## Abstract

**Simple Summary:**

Patients with locally advanced rectal cancer have been treated with chemoradiotherapy followed by surgery, which results in variable therapy response. To date, there is a lack of established predictive biomarkers to distinguish responsive from non-responsive patients. Therefore, patients can be overtreated resulting in unnecessary toxicity, or therapy changes can occur later when the cancer is more aggressive. We used pre-treatment biopsies to evaluate changes in DNA methylation that could predict the chemoradiotherapy response. Data from an external dataset were used to confirm our findings. We identified and validated a classifier, composed of three candidates, that was able to distinguish responders from non-responders. The genomic context of our biomarkers was explored, giving evidence that they play a role in regulating gene expression. The biomarkers herein described can be easily evaluated in the clinical practice and can help to guide rectal cancer patients’ treatment by identifying responders and non-responders.

**Abstract:**

The treatment for locally advanced rectal carcinomas (LARC) is based on neoadjuvant chemoradiotherapy (nCRT) and surgery, which results in pathological complete response (pCR) in up to 30% of patients. Since epigenetic changes may influence response to therapy, we aimed to identify DNA methylation markers predictive of pCR in LARC patients treated with nCRT. We used high-throughput DNA methylation analysis of 32 treatment-naïve LARC biopsies and five normal rectal tissues to explore the predictive value of differentially methylated (DM) CpGs. External validation was carried out with The Cancer Genome Atlas-Rectal Adenocarcinoma (TCGA-READ 99 cases). A classifier based on three-CpGs DM (linked to *OBSL1*, *GPR1*, and *INSIG1* genes) was able to discriminate pCR from incomplete responders with high sensitivity and specificity. The methylation levels of the selected CpGs confirmed the predictive value of our classifier in 77 LARCs evaluated by bisulfite pyrosequencing. Evaluation of external datasets (TCGA-READ, GSE81006, GSE75546, and GSE39958) reproduced our results. As the three CpGs were mapped near to regulatory elements, we performed an integrative analysis in regions associated with predicted cis-regulatory elements. A positive and inverse correlation between DNA methylation and gene expression was found in two CpGs. We propose a novel predictive tool based on three CpGs potentially useful for pretreatment screening of LARC patients and guide the selection of treatment modality.

## 1. Introduction

Patients with locally advanced rectal carcinomas (LARC) present a high risk of recurrence and death. Although these events can be reduced by preoperative neoadjuvant chemoradiotherapy (nCRT) followed by surgery, the treatment results in high morbidity, including severe side effects and high complication rates after surgery [1,2]. Patients with pathological complete response (pCR) to nCRT show lower rates of local and distant recurrences and better survival compared to patients with an incomplete response (pIR) [3]. Partial response to nCRT is observed from 70% to 90% of the patients, with about 20% showing resistance to treatment [4,5].

Several efforts have been conducted to precociously predict nCRT response [6,7,8,9,10], aiming to spare pCR patients of surgery [11,12], and to avoid unnecessary toxic exposure of radiotherapy in resistant patients [13,14]. To date, clinical examination alone is not able to accurately predict pCR. Although molecular markers have been described as potentially useful for this purpose, molecular screening analyses of LARC have shown conflicting results [15,16,17,18]. Gene expression signatures have been used to predict nCRT response; however, there are few or no overlapping genes amongst the published lists [19].

Disrupted DNA regulatory elements of gene expression can also be involved in the nCRT response and used as biomarkers [20]. Epigenetic alterations such as aberrant DNA methylation and covalent histone modifications are detected in all stages of cancer development. DNA methylation is considered an essential epigenetic mechanism involved in the regulation of gene expression [21]. DNA methylation-based biomarkers are useful tools to be applied in cancer diagnosis and prognosis. They are also associated with the therapeutic response and guide treatment decisions for cancer patients [22,23].

Genome-wide studies performed in different tumor types revealed methylome signatures able to stratify cancer subtypes [24], predict cancer outcomes [25,26,27,28], and identify epigenetic events related to inherent and acquired chemotherapy resistance [29]. Differential DNA methylation of specific CpG sites has contributed to the understanding of radiotherapy response with or without chemotherapy in a variety of cancer types, including glioblastoma, breast, gastric and colorectal cancers [30].

In LARC, DNA methylation assays have been described with distinct purposes, such as the identification of markers associated with the prognosis or response to therapy. *MGMT* (O6-methylguanine-DNA methyltransferase) promoter hypermethylation, the most known epigenetic alteration, was associated with better response to treatment in LARC patients [31]. Gaedcke et al. [32] identified 20 differentially methylated regions (DMR) in 11 LARC grouped according to the prognosis and validated the most significant 10 DMR in a larger cohort of cases (*n* = 113). The described classifier predicted the patient prognosis with a high grade of accuracy. Genome-wide DNA methylation was evaluated in 45 LARC samples classified according to the tumor regression grade (TRG) based on the Mandard classification [33]. The authors compared nCRT responders (TRG1-3) with non-responders [34]. From seven selected CpGs mapped in six genes (*DZIP1, ZEB1, DKK3, STL, KLHL34,* and *ARHGAP6*), only *KLHL34* methylation showed an association with response to nCRT. Nevertheless, increased methylation was not correlated with pCR.

The methylation landscape of LARC is poorly characterized, and yet to be explored. In this study, genome-wide DNA methylation analysis was performed in a carefully selected set of LARC cases to identify differences according to nCRT response. A classifier based on three CpGs was trained and validated in an independent group of cases. The correlation of the methylation levels of these three CpGs and gene expression and the respective neighboring enhancer interaction regions associated with predicted cis-regulatory elements were further investigated.

## 2. Results

### 2.1. Global DNA Methylation Profile of LARC Compared with Clinical Features and Gene Mutations

**Figure 1:**
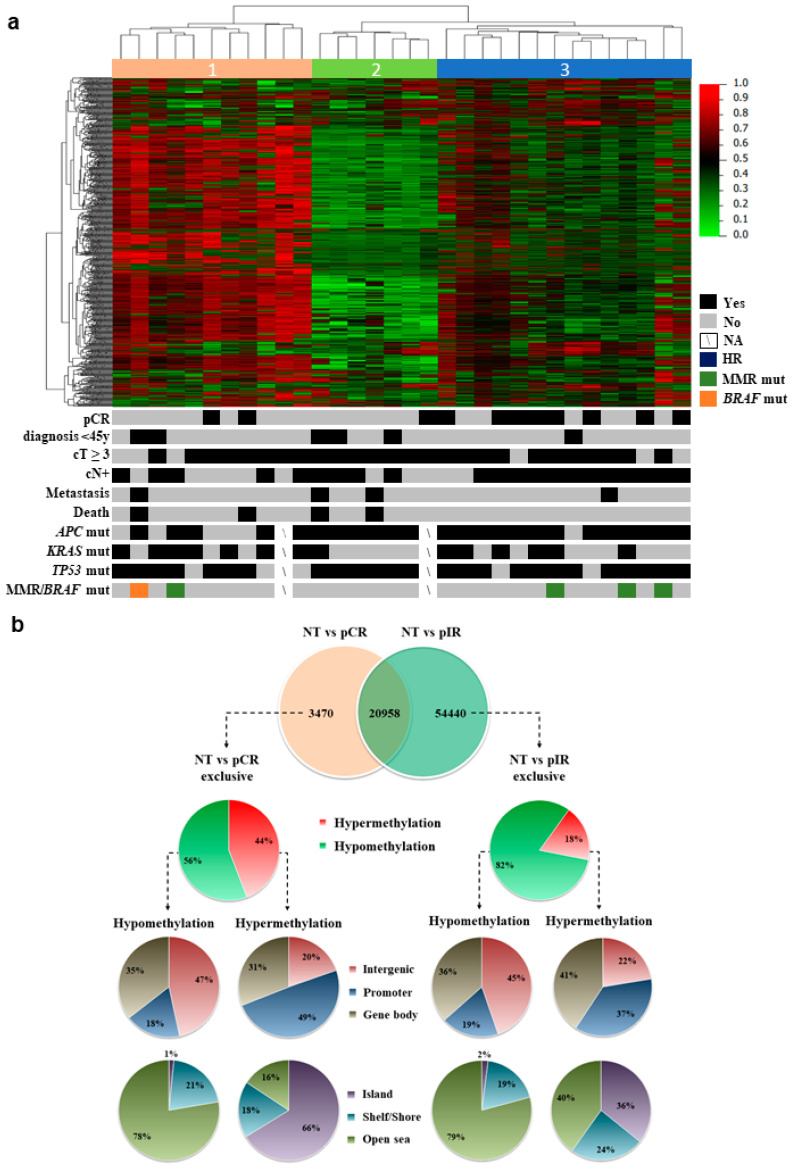
Methylation profile of locally advanced rectal carcinomas (LARC). (**a**) The unsupervised hierarchical clustering analysis of the methylation data using the most variable probes (6284 probes presenting β values with Standard Deviation >0.2) in the tumor samples revealed three major groups. Rows indicate the CpG sites, while columns represent samples. Clinical features of each case are represented below the heatmap along with targeted next-generation sequencing data for specific genes. Metastases were identified during the follow-up and after the treatment. (**b**) Distribution of the differentially methylated CpG probes in the comparison of pathological complete (pCR) or incomplete (pIR) response and normal tissue (NT) cases. The proportion of CpGs in relation to its location relative to the promoter, gene body, and intergenic regions; and to the CpG islands context was based on the Illumina EPIC annotation.

The genome-wide DNA methylation profiling (Infinium Methylation EPIC BeadChip microarray) was carried out in 32 LARC biopsies and 5 normal tissue (NT) samples. After filtering, 722,807 probes were normalized and used in the subsequent analysis. An unsupervised hierarchical clustering analysis comprising the most variable probes (6284 probes with standard deviation >0.2 in 32 LARC biopsies) revealed three distinct groups, with Cluster 3 being enriched with pCR cases (clusters 1 and 2: 3 pCR/18 LARCs and cluster 3: 8 pCR/14 LARCs; *p* = 0.0265, Fisher’s exact test) (Figure 1a). Other characteristics such as age or the presence of specific gene variants were not enriched in any of the clusters. One case harbored a variant in *BRAF* (c.1781A > G) and four cases in MMR genes (Figure 1a). Variants in *TP53* were almost ubiquitous (25/30 cases), and no significant association of pCR and cases with variants in *APC*, *TP53*, or *KRAS* was found, as we previously reported [35].

### 2.2. DNA Methylation Profile of LARC According to Pathological Response to nCRT and Comparison with the TCGA Dataset

**Figure 2:**
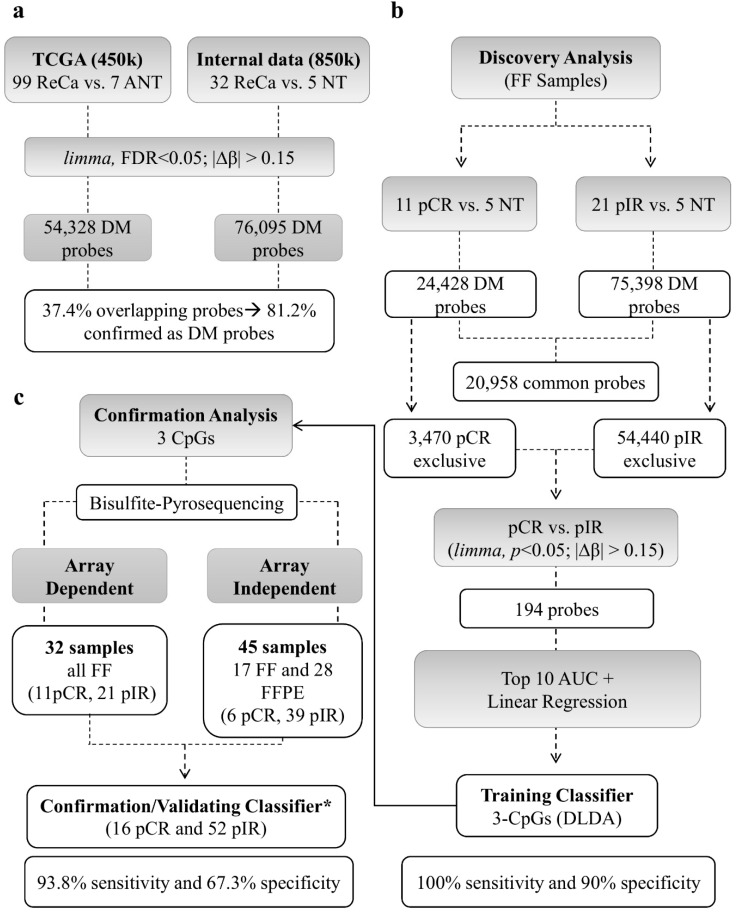
Sequential steps used to develop the DNA methylation classifier to predict neoadjuvant chemoradiotherapy (nCRT) response in rectal cancer (ReCa). (**a**) The methylation status of 99 ReCa and 7 adjacent normal tissue (ANT) samples from TCGA (Infinium Human Methylation 450K BeadChip platform, Illumina) was assessed for comparison. The identified differentially methylated (DM) probes were contrasted to those detected in our study (32 ReCa vs. 5 Normal tissues evaluated using the Infinium Human Methylation EPIC BeadChip platform–850K, Illumina), confirming 81.2% of DM probes in both studies (limma, False Discovery Rate < 5%; |∆β| > 0.15). (**b**) The ReCa cases were divided according to the response (11 pCR and 21 pIR) to nCRT to identify DM probes with potential predictive value. After filtering, a predictive classifier was trained by Diagonal Linear Discriminant Analysis (DLDA) using three probes (cg13770628, cg01072658, cg03085846). This classifier was able to distinguish pCR from pIR cases with 100% sensitivity and 90% specificity using the Leave-One-Out Cross-Validation (LOOCV) model. (**c**) The methylation status of these three probes was confirmed by bisulfite pyrosequencing in a set of 68 samples (32 array-dependent and 36 array-independent). * Only high-quality pyrosequencing results were included in the validation set. Sensitivity and specificity were calculated using LOOCV. FF: fresh frozen; FFPE: Formalin-fixed paraffin-embedded; DM: differentially methylated; ReCa: Rectal Cancer; NT: Normal Tissue; ANT: Adjacent Normal Tissue; pCR pathological Complete Response; pIR: pathological Incomplete Response.

The DNA methylation profile of LARC was compared to NT samples. Figure 2 summarizes the main results used to develop the DNA methylation classifier predictive of nCRT response. A total of 76,095 DM probes (limma, False Discovery Rate - FDR < 5%; |∆β| > 0.15) was detected, of which 59,044 CpGs were hypomethylated (78%) and 17,051 hypermethylated (22%) in tumors (Supplementary Materials Table S1). We compared our DM probes (EPIC BeadChip–850k array) with The Cancer Genome Atlas Rectal Adenocarcinoma (TCGA-READ) dataset (99 Rectal Cancer–ReCa, versus 7 adjacent normal tissue–ANT) derived from the Infinium HumanMethylation 450k BeadChip. The TCGA analysis resulted in 54,328 DM probes (33,297 hypomethylated and 21,031 hypermethylated probes) (Figure S1). A total of 37.4% probes from our study overlapped with those from the TCGA dataset and 81.2% of them were confirmed as DM (Figure 2a).

The comparison between pCR and pIR versus NT revealed 24,428 and 75,398 DM probes, respectively. Among these probes, 20,958 were DM in both groups, while 3470 probes were DM only in pCR and 54,440 in pIR (Figure 2b). The DM probes unique to each group were used to better characterize the methylome of LARC according to the nCRT response. Tumors from pCR patients presented a higher proportion of hypermethylated probes (44% compared to 18% in pIR), which were mainly located in the promoter regions (49% vs. 37% in pIR) and CpG islands (66% vs. 36% in pIR), while biopsies from pIR patients predominantly showed a hypomethylated profile (82% of the DM CpGs) (Figure 1b).

### 2.3. Three Hypomethylated CpGs are Predictive of nCRT Response in LARC

**Figure 3:**
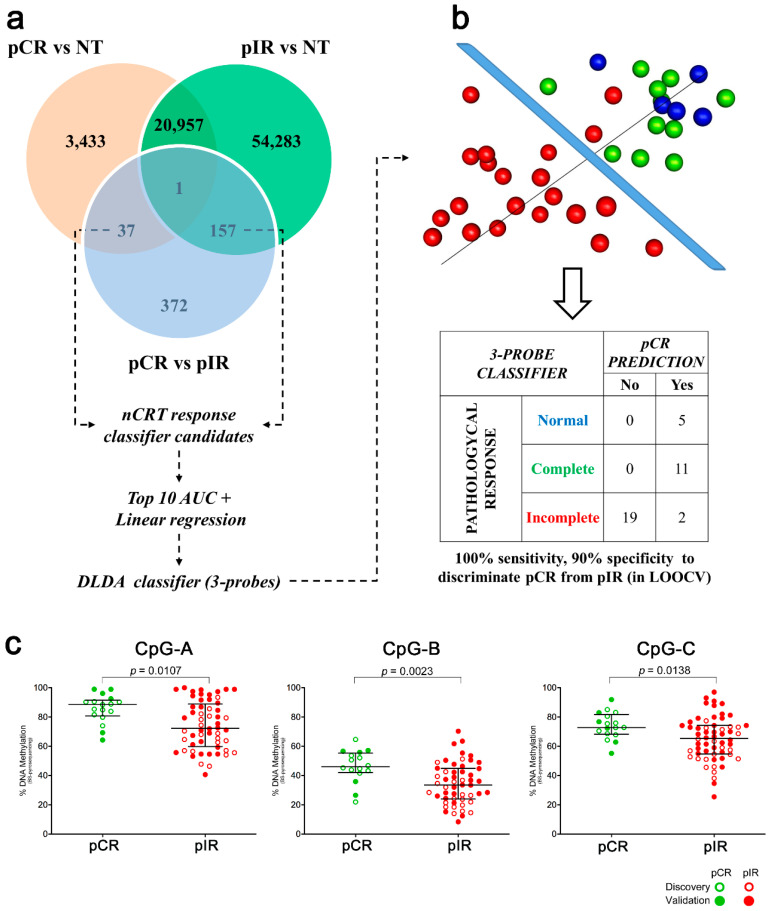
Development and performance of the classifier associated with response to treatment. (**a**) Venn diagram showing the number of differentially methylated probes overlapping among the group comparisons (pCR vs. NT, pIR vs. NT, and pCR vs. pIR) that were used for developing a predictive model based on Diagonal Linear Discriminant Analysis (DLDA). (**b**) Tridimensional distribution of samples according to the methylation values of the three probes included in the classifier and their performance in discriminating patients with pCR or pIR tested using Leave-One-Out Cross-Validation (LOOCV). Normal tissue (NT) samples were also tested using the classifier. (**c**) Methylation status of three CpGs (CpG-A: cg01072658, CpG-B: cg03085846, CpG-C: cg13770628) identified as potential biomarkers of response to neoadjuvant chemoradiotherapy in locally advanced rectal cancer evaluated by bisulfite pyrosequencing. BS-pyroseq: bisulfite pyrosequencing, pCR: pathological complete response, pIR: pathological incomplete response.

The assessment of locus-specific altered by methylation in treatment-free biopsies is a potential tool to unravel biomarkers of nCRT response. To identify these candidates, we selected the exclusive DM probes of each group (3470 and 54,440 CpGs in pCR and pIR groups, respectively) and between both groups (194 probes in pCR vs. pIR) (Figure 3a; Table S2). Next, the probes were filtered based on the highest area under the receiver operating characteristic (ROC) curves (AUC) and linear regression analysis. Three hypomethylated CpGs (CpG-A: cg01072658, CpG-B: cg03085846, and CpG-C: cg13770628) predictive of nCRT response were trained by DLDA (Score = CpG-A X − 19.3 + CpG-B X − 11.2 + CpG-C X − 24.6; pathological response prediction threshold > −35.9). The classifier was able to distinguish 11 pCR from 21 pIR cases with 100% sensitivity and 90% specificity using the LOOCV approach (Figure 3b). Five NT samples tested using this classifier were categorized as pCR.

### 2.4. Bisulfite Pyrosequencing Analysis Confirmed the Performance of the Classifier

**Figure 4:**
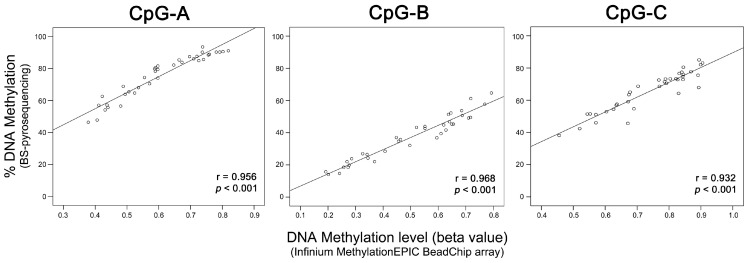
Correlation between the methylation levels of the three CpGs of LARC samples determined by microarray (Infinium MethylationEPIC BeadChip array, Illumina) and bisulfite (BS) pyrosequencing analysis. The scatterplots show a high positive correlation for three CpGs identified as a predictive classifier of response to neoadjuvant chemoradiotherapy. (*r*) Pearson’s correlation coefficient, (*p*) *p*-value from Pearson’s correlation test.

To evaluate the performance of the three-CpGs DNA methylation-based classifier, BS-pyrosequencing was performed in 77 LARC (32 from discovery and 45 from validation set) and five NT samples (Figure 2c). DNA methylation levels determined by BS-pyrosequencing and methylation microarray for each CpG-A, CpG-B, and CpG-C showed high concordance (*r* = 0.956, *r* = 0.968, and *r* = 0.932, respectively, *p* < 0.001 for all comparisons) (Figure 4).

Pyrosequencing yielded good quality results for 5 NT samples and 73, 69, and 77 LARC for CpG-A, CpG-B, and CpG-C, respectively. There was no difference in the methylation values of FFPE (Formalin-Fixed Paraffin-Embedded ) and FF (Fresh Frozen) samples for the CpGs-B and C, but higher methylation in the FFPE samples for the CpG-A (Figure S2a) was found. The comparison between the methylation levels in the discovery and validation samples showed no difference (Figure S2b).

All three CpGs were hypomethylated in pIR compared to pCR (*p* < 0.05) (Figure 3c), and their AUC showed promising results as predictive markers (AUC: CpG-A = 0.706; CpG-B = 0.754; and CpG-C = 0.697) (Figure S3). The classifier based on three CpG (pyrosequencing classifier threshold adjusted to −3621.9) was able to distinguish pCR (*n* = 16) from pIR (*n* = 52) with 93.8% sensitivity and 67.3% specificity.

### 2.5. Prediction of the Impact of DNA Methylation Changes of the CpG-A, CpG-B, and CpG-C on Gene Expression in LARC

Combining DNA methylation and gene expression can bring insights to explain the predictive impact on the response to therapy of LARC patients. We integrated the DNA methylation used in the discovery phase of our study with our previous study of gene expression levels (GSE123390) of 27 LARC [36]. The CpG-A, CpG-B, and CpG-C are associated with *OBSL1* (Obscurin Like Cytoskeletal Adaptor 1), *GPR1* (G Protein-Coupled Receptor 1), and *INSIG1* (Insulin-induced gene 1) loci, respectively. The inferred target genes mapped across the predicted interaction regions were further investigated in pCR and pIR groups.

**Figure 5:**
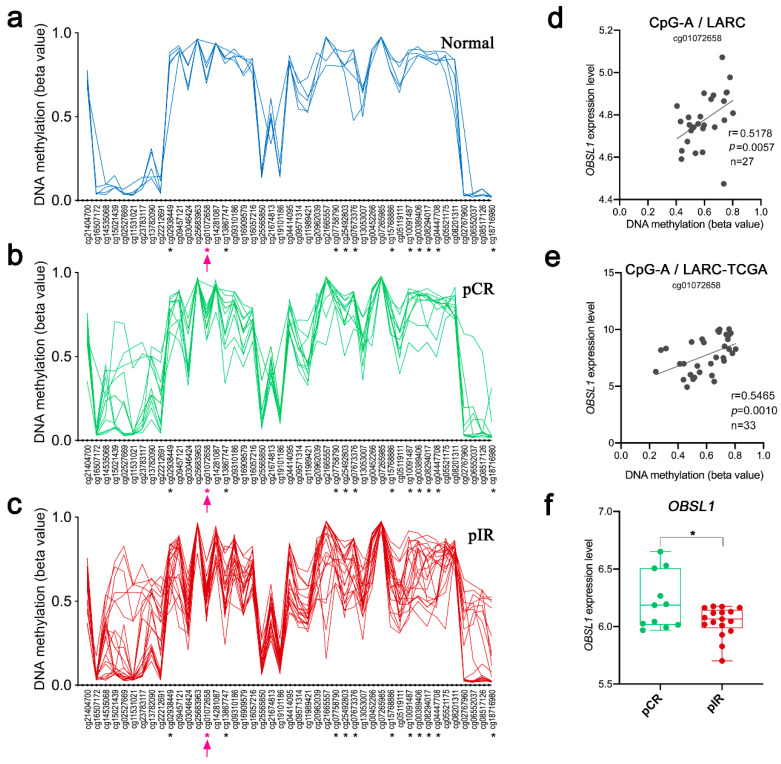
DNA methylation levels of 45 CpGs mapped in the region of the *OBSL1* gene in (**a**) normal rectal tissue and LARC samples classified according to nCRT response (**b)** pCR—complete response and (**c**): pIR—incomplete response). The symbol * indicates significant differences between the groups; the CpG-A (cg01072658) is highlighted in pink. (**d**) Correlation between the DNA methylation levels of the CpG-A and the *OBSL1* gene expression levels in 27 LARC from our cohort and (**e**) in a set of LARC from TCGA-READ data collection. (**f**) The expression levels of the *OBSL1* gene were lower in the pIR group (*p* < 0.05).

The CpG-A is mapped within the gene body of *OBSL1*. This gene is associated with a distal cis-regulatory element (regulatory enhancer element for *OBSL1* gene/GH02J218908 located at 648 kb upstream to CpG-A) that presents multiple predicted interactions across a region spanning ~0.66 Mb on chromosome 2 (nucleotide position of the target region, chr2:220,436,581–220,436,581). This genomic region contains 25 predicted target genes, 276 inferred transcription factor (TF) binding sites, and it is represented by 880 CpGs in the Infinium MethylationEPIC 850k array, 50 of them identified as DM in our LARC cases. This target region is characterized by focal hypermethylation segments embedded in long-range regions of hypomethylation (Figure S4a,b,d). The *OBSL1* gene is covered by 45 gene-body probes, 12 of which are DM, including the CpG-A and 11 other CpGs that presented lower levels of DNA methylation in pIR compared with pCR (Figure 5a–c). The CpG-A hypomethylation was significantly correlated with *OBSL1* gene expression in 27 LARC samples (Spearman *r* = 0.5178, *p* = 0.0057) (Figure 5d). This finding was confirmed in an independent set of 33 LARC samples from the TGCA-READ dataset (Spearman *r* = 0.5465, *p* = 0.0010) (Figure 5e). Decreased *OBSL1* gene expression levels were found in pIR compared to pCR cases (Figure 5f). The expression levels of eight genes mapped on the enhancer interaction region were also significantly different between pCR and pIR groups: while *CNOT9*, *ZNF142*, and *TTLL4* showed increased expression, the genes *PLCD4*, *PRKAG3*, *CFAP65*, *NHEJ1*, and *GBL1* were downregulated in pIR compared to pCR cases (Figure S4c). Only two DM CpGs (cg09791753 and cg14909250) were associated with the *GBL1* gene. The cg09791753 is located within a CpG island spanning the transcription start site (TSS), and the 5’UTR of the *GBL1* gene and its methylation level was negatively correlated with the gene expression levels (Spearman *r* = −0.4658, *p* = 0.0143).

The CpG-B is located at 2175 pb and 132.4 Kb upstream to two enhancer regulatory elements associated with the *GPR1* gene (GH02J206211, nucleotide position chr2:207,076,106–207,078,770 and *ZDBF2*/GH02J206081 nucleotide position chr2:206,945,799–206,952,525). The *GPR1* interaction region is associated with 56 inferred TF binding sites and three predicted target genes. The region overlapping with the enhancer’s target region for *ZDBF2* gene (*ZDBF2*/GH02J206081) is associated with 243 inferred TF binding sites and ten predicted target genes. The expression levels of the *SNORD51* gene and the antisense long non-coding RNA (lncRNA) *GPR1-AS* were downregulated in pIR compared to pCR group (Figure S5c). However, the methylation level of CpG-B (intronic to the lncRNA *GPR1-AS*) was not correlated to the *GPR1* and *GPR1-AS* expression levels.

The CpG-C is the only DM CpG located at 3554pb downstream to the regulatory enhancer element for the *INSIG1* gene (GH07J155282, nucleotide position chr7:155,074,428–155,074,660) and 11,504 pb upstream to the TSS of this gene. The predicted interaction region contains four genes and one cis binding site for the transcription factor *EBF1*. The DNA methylation changes of CpG-C are negatively correlated with the *INSIG1* gene expression (Spearman r = −0.4249, *p* = 0.0153). *INSIG1* gene expression levels were higher in pIR compared to pCR patients (Figure S6).

## 3. Discussion

The highly variable response to nCRT in rectal cancer requires a better understanding of the disease and the identification of biomarkers capable of precociously stratifying patients according to the treatment response. The DNA methylation profile of these tumors has not been thoroughly explored, and to our knowledge, only one study has evaluated the global methylation status of LARC concerning the response to nCRT [34].

We initially compared the overall methylation status of ReCa in relation to normal tissues found in our study with the data available in public databases [37,38]. To gain insights on the methylation profile of LARC, the probes were classified according to their genomic context as CpG islands, shores, shelves, or open sea [39]. In agreement with two previous studies [37,38] that used the Illumina 450K platform, we found high levels of hypomethylation, especially in intergenic regions and open sea, while hypermethylation was mostly detected within promoters and CpG islands. In 25 ReCa paired with adjacent normal tissues, Vymelkova et al. [40] reported 5929 DM CpGs with a prevalence of hypomethylation in intergenic and open sea regions and CpG islands hypermethylation. These findings demonstrate the reproducibility of our results and corroborate the previous data on the DNA methylation profile of ReCa.

We showed a higher proportion of hypermethylated probes in pCR (44%) compared to pIR (18%). The hypomethylated probes presented similar distribution throughout the genome, while the hypermethylated probes were mostly detected in CpG islands in pCR (66%) compared to pIR (36%). Overall, these findings support that ReCa is characterized by focal hypermethylation segments embedded in long-range regions of hypomethylation, as previously reported in colorectal cancer (CRC) [41]. Parallel analysis with the previously published study in DNA methylation profile in LARC and response to nCRT [34] was precluded because the authors did not compare tumors with NT.

Most of the studies describing DNA methylation in LARC evaluated a small panel of genes as biomarkers of nCRT response. Hypermethylation of *ATM* (ATM serine/threonine kinase)*, CRBP1* (RBP1 retinol binding protein 1)*, MGMT, TFAP2E* (transcription factor AP-2 epsilon), and *TIMP3* (metallopeptidase inhibitor 3) genes was associated with response to radiotherapy and/or 5-FU (5-fluorouracil) based chemotherapy in ReCa [17,20,31,42,43]. In our study, five probes hypermethylated in *TFAP2E* and one hypomethylated in *TIMP3* were found in both groups (pCR and pIR) compared to NT. In circulating tumor cells, *MGMT* promoter hypermethylation was associated with a better response to nCRT [31]. Contrarily, we identified DM probes in CpGs within the *MGMT* gene body in pIR cases. Although alteration in gene expression due to DM regions in gene bodies has been previously reported [44,45], we found no correlation of the abovementioned DM probes and *MGMT* expression, evaluated in our previous transcriptomic data performed in the same group of cases (GSE123390) [36].

We built a classifier predictive of nCRT response based on the DNA methylation levels of three CpG dinucleotides. Subsequent analyses of these three CpGs using BS-pyrosequencing showed a high positive correlation with the DNA methylation values determined by microarray analysis. These three CpGs were confirmed as DM in pCR compared to pIR in array-dependent and independent samples, reinforcing their relevance in predict the response to therapy in LARC.

Four datasets presenting available data on rectal tumors (TCGA-READ, GSE75546, and GSE39958) or CRC cell lines treated with 5-FU (GSE81006) were assessed to verify the methylation pattern of our three CpGs. These studies used the Infinium Human Methylation 450K BeadChip platform (Illumina), and only the CpG-A (cg01072658) was observed in both platforms (850k platform used in our study). Lower methylation values for CpG-A (*p* < 0.01) (Figure S7a), and a significant correlation with *OBSL1* gene expression was observed in the TCGA cohort (Figure 5e). Similarly, the methylation levels of this probe in the GSE75546 study were lower in six LARC compared to paired ANT samples (Figure S7b) [37]. The GSE39958 study described high-throughput DNA methylation analysis of 45 LARC associated with response to nCRT [34] based on the Mandard et al. [33] classification system. However, no difference was observed in the CpG-A probe methylation regarding the response to therapy (7 cases TRG1 and 38 TRG2-5, Figure S7c). Of note, two TRG1 cases presented much lower methylation levels than all pCR cases from our cohort, strengthening the need to evaluate this CpG in a larger group of cases.

Shen and colleagues [46] have established a 5-FU resistant cell line from its parental wild type CRC HCT-8 cell line. The authors treated both cell lines with 5-FU for 0 and 72 h and evaluated three replicates of each condition using the Illumina 450k Methylation Beadchip (GSE81006). The methylation status of the CpG-A probe in resistant cells was significantly lower compared to wild type cells (Figure S7d), suggesting that this CpG could be a useful marker of 5-FU response. This finding reinforces the involvement of the CpG-A as a marker of nCRT response.

Interestingly, the three CpGs of the classifier are located in known regulatory elements. The CpG-A is mapped in the *OBSL1* gene body, CpG-B is located in the lncRNA *GPR1-AS* (GPR1 antisense RNA), and the CpG-C is within the intergenic region of chromosome 7. Therefore, the surrounding CpGs and the genes predicted as regulated by these regions were further investigated. We identified 50 DM CpGs flanking the CpG-A, 10 of which were associated with the *OBSL1* gene (Figure 5). Nine genes (including the *OBSL1*) were differentially expressed between pCR and pIR. These genes were predicted as regulated by the *OBSL1* enhancer region, which presented a significant correlation to the methylation levels of the CpG-A. The *OBSL1* gene encodes a component of the 3M complex, which is required to regulate microtubule dynamics and genome integrity [47]. It is well known that genomic instability is one of the hallmarks of cancer development [48], and many studies have also shown its association with response to therapy [49]. However, the specific involvement of *OBSL1* in cancer is insufficiently explored. Only an association with the endocytosis pathway of human papillomavirus (HPV) has been reported [50].

The region involving the CpG-B also presented four other DM probes. The expression of *GPR1*-AS and *SNORD51* (small nucleolar RNA, C/D box 51) predicted as regulated by the same enhancer was significantly different between pCR and pIR. The function of *GPR1-AS* is currently unknown, but this lncRNA was shown to be located within an imprinted region, expressed only in the placenta, with biallelic promoter hypermethylation in adult tissues [51]. The consequences of changes in DNA methylation or expression patterns of this lncRNA has not been reported.

The CpG-C is mapped within the *INSIG1* enhancer interaction region, with no other DM CpG in the flanking regions. However, a significant negative correlation was found between the beta values of CpG-C and the *INSIG1* gene expression (Figure S6c). This gene presented higher expression levels in pIR compared to pCR cases (Figure S6d). *INSIG1* has been implicated in epithelial-mesenchymal transition, which is also one of the hallmarks of cancer [52,53]. Besides, its increased expression seems to be stimulated by cell-free DNA from colorectal cancer cells [54], and by treating cell lines with chemotherapy agents. Increased expression of the *INSIG1* gene was described in breast cancer cell lines after treatment with gemcitabine and 5–FU [55], while decreased expression was detected after treating a laryngeal cancer cell line with low doses of paclitaxel [56], and after anacardic acid treatment in breast cancer cell lines [57].

We verified that most of the DM CpGs mapped in regulatory regions were hypomethylated, including the three-CpG identified as predictive of nCRT response. It is known that DNA hypomethylation and active enhancer signatures show a high degree of cell-type specificity [58]. Therefore, we used the available data of the chromatin states of two normal rectal mucosae [59] to identify active genes overlapping the DM probes and the active TSS across the enhancer region for the *OBSL1* gene (Figure S4a). Among the eight inferred genes differentially expressed in pCR and pIR cases, two DM CpGs were associated with the *GLB1L* (galactosidase beta 1 like) gene, and only one showed correlation with its expression level. It is notorious that the network among DNA methylation, cis-regulatory elements, and the variety of potential TFs involved in this process is complex. The underlying mechanisms by which alterations in specific CpGs affects gene expression are not thoroughly unveiled. While the correlation between gene expression and DNA methylation in promoter regions is well described and explored, the effect of DNA methylation in other genomic regions remains poorly understood. A positive correlation, providing evidence of a causal association between gene body methylation and gene expression, was described in cancer [44,60]. However, subsequent studies demonstrated that DM CpGs located in the gene body could have both positive and negative correlation with gene expression, disclosing other regulatory mechanisms [61,62]. The impact of DNA methylation changes on differential gene expression between pCR and pIR herein reported could be due to an indirect effect in which DNA methylation avoid the binding of TF to its cognate site, or the TFs are drivers of the hypomethylated state [63]. Although these mechanisms need to be better evaluated in the context of response to treatment, our findings suggest that DNA methylation changes of the three-CpGs are implicated in the nCRT response in LARC.

Our results demonstrate that the methylation status of three CpGs is a potential predictive tool for nCRT response. Although promising, some limitations need to be considered. We used normal tissues obtained from cancer-free individuals (autopsies) for comparison to LARC biopsies, while others used adjacent normal tissues. Several concerns have been reported in using surrounding normal tissues as a control to identify molecular alterations in tumors. It has been demonstrated that ANT presents characteristics that differentiated them from healthy tissues [64]. We used FFPE and FF samples in our confirmation dataset, which could be questioned. For instance, one of the CpGs (CpG-A) showed higher methylation levels in FFPE compared to FF samples. However, we observed significant hypomethylation in pIR cases indicating that our results are not biased by sample preservation. The platform used in our study interrogates the methylation status of almost twice as many probes used elsewhere, which brings new information about these tumors but restricts the number of studies available for comparison and data confirmation.

In the present study, we described a classifier composed of three differentially methylated CpGs able of distinguishing LARC cases according to nCRT response, which has the potential to be used in the clinical practice.

## 4. Materials and Methods

### 4.1. Patients

We selected strictly 53 patients of a large retrospective cohort of 556 diagnosed with LARC (based on the American Joint Committee on Cancer 7th Ed.) [65] admitted at A.C. Camargo Cancer Center and Barretos Cancer Hospital, São Paulo-Brazil, between 2006 and 2015. The biopsies were collected during the colonoscopy and before nCRT. All patients were referred to nCRT with continuous infusion of 5-FU (fluorouracil) or oral capecitabine and radiotherapy (total dose of 50.4 Gy) followed by surgery. Further eligibility criteria include no other cancer and/or metastases at diagnosis, biopsies available in the biobanks, and complete surgical histopathological information. A cohort of 24 patients treated at the University Hospital of Southern Denmark, Vejle, DK, between 2016 and 2020, was recruited following the same inclusion/exclusion criteria,. Thirty-two LARC cases were used for a discovery screening and 45 for data confirmation. Twenty-eight FFPE out of these 45 tumor samples were included due to the difficulty of finding available FF biopsies.

The pathological response to the nCRT, defined as the absence or presence of reminiscent viable tumor cells in the surgical specimens on hematoxylin and eosin evaluation, was used to classify the patients into two groups: pCR (ypT0N0) or pIR. Clinical and epidemiological data are summarized in Table 1. Histopathological normal rectal tissues were also collected from five individuals with the same median age (autopsies). The study was conducted in accordance with the ethical guidelines and regulations of the Declaration of Helsinki. The Ethics Committees from A.C. Camargo Cancer Center (#1884/14), Barretos Cancer Hospital (#1030/2015), Regional Committee on Health Research Ethics of Southern Denmark, and the Danish Data Protection Agency (Protocol # S20160097) approved the study. Written informed consent was obtained from all patients or family members before sample collection.

### 4.2. DNA Isolation and Bisulfite Conversion

Genomic DNA was extracted from 54 FF (49 tumors LARC and 5 NT) tissue specimens using phenol-chloroform-isoamyl alcohol (25:24:1) solution-based protocol, and 28 FFPE tumor specimens using RecoverAll™ Total Nucleic Acid Isolation Kit for FFPE (ThermoFisher, Waltham, MA, USA). The DNA was quantified using Qubit® dsDNA BR Assay (Life Technologies, Carlsbad, CA, USA). Bisulfite conversion was performed with the EZ DNA Methylation-Gold™ Kit (Zymo Research, Irvine, CA, USA), according to the manufacturer recommendations.

### 4.3. High-Throughput DNA Methylation Profiling

The genome-wide DNA methylation profiling of 32 tumor biopsies (discovery set: 11 pCR and 21 pIR) and five NT samples was assessed using the Infinium Human MethylationEPIC BeadChip platform (Illumina, San Diego, CA, USA), according to the manufacturer recommendations. The large-scale methylation data is available at the Gene Expression Omnibus database (GSE132668 https://www.ncbi.nlm.nih.gov/geo/). The original data can also be obtained from the authors upon request. The microarrays were scanned using the Illumina HiScan system, and the data were processed using R language. Briefly, quality control, p-value detection for all probes, background noise detection, and adjustment were performed using the package minfi [66]. The Beta MIxture Quantile dilation (BMIQ) model was used to adjust for differences of Type-I and Type-II probes and to normalize the β values [67]. Batch effects were removed using the package SVA [68]. Probes with low quality (detection *p*-value > 0.05), mapped in X/Y chromosomes, and cross-reactive were filtered out, as previously described [69]. Probes mapped to Single Nucleotide Polymorphisms were removed using the package minfi [66]. Unsupervised hierarchical clustering analysis was performed using BRB array tools v.4.4.0 (Biometric Research Branch, National Cancer Institute, USA—https://brb.nci.nih.gov/BRB-ArrayTools/index.html). Differentially methylated (DM) probes, contrasting pCR or pIR and NT, were identified using the limma package [70]. Probes with FDR < 5% and |∆β| > 0.15 were considered significant and annotated using the Illumina manifest file. The study design is illustrated in Figure 2.

### 4.4. DNA Methylation-Based Algorithm Predictive of Response to Neoadjuvant Treatment in LARC

The DM CpGs identified exclusively in pCR or pIR cases and also different between them (limma *p* < 0.05; |∆β| > 0.15) were used to develop a predictive classifier. The most informative probes in distinguishing pCR from pIR were filtered based on the AUC (top 10 higher AUC). Redundant markers were eliminated using linear regression analysis (stepwise selection method). The potential CpG markers were submitted to a Diagonal Linear Discriminant Analysis (DLDA), and the classifier performance was estimated using Leave-One-Out Cross-Validation (LOOCV) (BRB array tools v.4.4.0).

### 4.5. Data Confirmation by Quantitative Bisulfite Pyrosequencing

Seventy-seven LARC biopsies (17 pCR and 60 pIR cases) were evaluated using bisulfite (BS)-pyrosequencing to confirm the DNA methylation levels of the selected CpGs. Primers sequences and PCR conditions used in the BS-pyrosequencing for each CpG (cg01072658, cg03085846, and cg13770628) are described in Table S3. After bisulfite conversion, 10ng of DNA was amplified using the PyroMark PCR kit (Qiagen, Germantown, MD, USA), according to the manufacturer protocol. The PCR products were sequenced on the PyroMark Q24 system (Qiagen, Germantown, MD, USA).

To corroborate the performance of the response predictive model designed using the large-scale method, the same mathematical model was adopted in the BS-pyrosequencing analysis. However, because of the different quantification scales obtained by microarray and BS-pyrosequencing, the threshold was adjusted to achieve the best overall accuracy.

### 4.6. External Data Analysis

Methylation data from TCGA-READ (7 ANT and 99 ReCa) were downloaded from UCSC Xena repository (http://xena.ucsc.edu, last accessed 6 May 2020). The DM probes (FDR < 5% and |∆β| > 0.15) were used for confirming the ReCa global methylation profile. Specific CpGs were investigated in a subgroup of 38 LARC from TCGA-READ cases. The beta values from studies reporting DNA methylation data of LARC (GSE75546, and GSE39958) [34,37] and colorectal cell lines (GSE81006) [46] were downloaded from the Gene Expression Omnibus (GEO, https://www.ncbi.nlm.nih.gov/geo/) and analyzed using GraphPad Prism 8.0 (GraphPad Software Inc., San Diego, CA, USA). Targeted next-generation sequencing data of a panel with 105 cancer-related genes from 30 of the 32 LARC cases were retrieved from our previous study [35] (deposited at Sequence Read Archive–SRA: PRJNA535396) to assess variants in key CRC-related genes. To evaluate the correlation between CpG-specific methylation levels and gene expression, we used the matched microarray-based transcriptomic data from 27 of 32 LARC cases previously reported by our group (GSE123390) [36]. To further explore these correlations in an independent set of LARC cases, we retrieved the matching methylation (Infinium HumanMethylation450k BeadChip) and transcriptomic (RNA-Seq) data publicly available from 33 LARC cases from TCGA-READ study using the Wanderer interactive online tool (http://maplab.imppc.org/wanderer/) [71].

### 4.7. Integrative Analysis of the Predicted Cis-Regulatory Elements Associated with the Flanking Regions of the Predictive Classifier

A detailed in silico analysis was performed to gain a comprehensive understanding of the impact of the DNA methylation changes of the three CpGs (CpG-A: cg01072658, CpG-B: cg03085846, and CpG-C: cg13770628) on gene expression. The sequence of these probes was aligned with the human reference genome (Genome Reference Consortium Human Build 37—GRCh37/hg19, UCSC Genome Browser platform at https://genome.ucsc.edu/). The genome location and epigenetic context were evaluated by overlapping these probes with predicted cis-regulatory elements such as promoter-associated CpG islands, distal enhancers, and DNA motifs harboring TF binding sites. The clustered interactions of predicted regulatory elements and inferred target genes by GeneHancer (GeneHancer database, available at http://www.genecards.org/) was used to integrate DNA methylation, chromatin status, and gene expression data (Appendix A).

### 4.8. Statistical Analysis

Statistical analyses were performed using R (v.3.5.1), SPSS (SPSS v. 21.0, IBM - Armonk, NY, USA), BRB array tools v.4.4.0 (Biometric Research Branch, National Cancer Institute, Bethesda, MD, USA–https://brb.nci.nih.gov/BRB-ArrayTools/index.html), and GraphPad Prism 8.0 (GraphPad Software Inc., San Diego, CA, USA). The methylation values of each CpG assessed by microarray and BS-pyrosequencing were compared using the Pearson correlation test. Mann-Whitney test was used to evaluate the differences between methylation values. Fisher exact test was applied to verify the distribution of the values between the two groups. The ability to predict response to nCRT of each marker was verified by the AUC. The correlation between the beta-values of each of the three CpGs and gene expression was calculated using the Spearman’s correlation test. Statistical significance was considered with *p* < 0.05.

## 5. Conclusions

We identified and confirmed that three differentially methylated CpGs were capable of classifying LARC cases according to nCRT response. The methylation assessment of these genomic regions can be performed in a clinically compatible setting, using a low-cost technique such as BS-pyrosequencing. We also identified the regulatory regions associated with each CpG, suggesting their role in regulating gene expression.

## Figures and Tables

**Table 1:** Clinical and histopathological features of locally advanced rectal cancer patients included in this study.

Characteristics	Number of Patients (Total: 77)
Median age at diagnosis	60
Gender	
Female	45
Male	32
Response to Neoadjuvant therapy	
pCR	17
pIR	60
cT stage	
Tx	1
T2	9
T3	57
T4	10
cN stage	
N0	20
N+	57
ypT stage	
T0	19
T1	5
T2	24
T3	25
T4	4
ypN stage	
N0	55
N+	22

TNM: tumor-node-metastasis clinical (c) and histopathological assessment after neoadjuvant treatment (yp) for classification of malignant tumors (American Joint Committee on Cancer 7th edition [65]), pCR: pathological complete response, pIR: pathological incomplete response.

## Supplementary Materials

The following are available online at https://www.mdpi.com/2072-6694/12/11/3079/s1, Table S1: Probes differentially methylated (76,095) in locally advanced rectal cancer compared to normal tissues (limma *p* < 0.001; FDR < 5%; |Δβ| > 0.15) (EPIC BeadChip platform, Illumina), Table S2: Candidate probes (*n* = 194) used to predict pathological complete response to neoadjuvant chemoradiotherapy in locally advanced rectal carcinomas, Table S3. Primer sets used in the pyrosequencing reaction for three CpGs that compose the predictive classifier of neoadjuvant chemoradiotherapy response in locally advanced rectal cancer, Figure S1: Schematic representation of the genomic distribution and the CpG context of the differentially methylated (DM) probes identified in rectal cancer (ReCa) samples in the internal and external (The Cancer Genome Atlas, TCGA, and Wei et al., 2016) datasets compared to non-neoplastic rectal mucosa samples, Figure S2: Quality control assessment of the samples evaluated by pyrosequencing, Figure S3: Performance of the three CpGs in discriminating response to neoadjuvant treatment in rectal cancer patients (pCR versus pIR),Figure S4: Genomic context and expression of the genes involved in the expanded interaction region of *OBSL1* enhancer, Figure S5: Physical map of the expanded interaction region of *GPR1* enhancer, Figure S6: Physical map of the interaction region of the enhancer *INSIG1*, Figure S7: External independent datasets containing the methylation status of the CpG-A (cg01072658 probe, Infinium Human Methylation 450k BeadChip platform, Illumina) in rectal cancer samples or colorectal cancer cell lines sensitive (HCT8_WT) or resistant (HCT8_resistant) to 5-fluorouracil (5-FU).

## Appendix A

Supplementary methods describing the analyses of the epigenomic landscape of the neighboring regulatory regions to the CpG-A, CpG-B, and CpG-C.
